# Synthesis, Structure and Thermal Behavior of Oxalato-Bridged Rb^+^ and H_3_O^+^ Extended Frameworks with Different Dimensionalities

**DOI:** 10.3390/ma3021281

**Published:** 2010-02-23

**Authors:** Hamza Kherfi, Malika Hamadène, Achoura Guehria-Laïdoudi, Slimane Dahaoui, Claude Lecomte

**Affiliations:** 1 Laboratoire de Cristallographie-Thermodynamique, Faculté de Chimie, USTHB, BP 32, El-Alia, Bab-ezzouar, Algiers, Algeria; E-Mails: khe_hamza@hotmail.com (H.K.); mal_hamadene@hotmail.com (M.H.); 2 CRM2, UMR-CNRS, 7036, Institut Jean Barriol, Nancy Université, BP239, 54506, Vandoeuvre-lès Nancy Cedex, France; E-Mails: slimane.dahaoui@crm2.uhp-nancy.fr (S.D.); claude.lecomte@crm2.uhp-nancy.fr (C.L.)

**Keywords:** extended framework, thermal behavior, hydrogen-bonded motifs, hybrid materials, cocrystals, coordination polymers, supramolecular synthesis, ferroïc properties, homosynthon

## Abstract

Correlative studies of three oxalato-bridged polymers, obtained under hydrothermal conditions for the two isostructural compounds {Rb(HC_2_O_4_)(H_2_C_2_O_4_)(H_2_O)_2_}_∞_^1^, **1**, {H_3_O(HC_2_O_4_)(H_2_C_2_O_4_).2H_2_O}_∞_^1^, **2**, and by conventional synthetic method for {Rb(HC_2_O_4_)}_∞_^3^, **3,** allowed the identification of H-bond patterns and structural dimensionality. Ferroïc domain structures are confirmed by electric measurements performed on **3**. Although **2** resembles one oxalic acid sesquihydrate, its structure determination doesn’t display any kind of disorder and leads to recognition of a supramolecular network identical to hybrid s-block series, where moreover, unusual H_3_O^+^ and NH_4_^+^ similarity is brought out. Thermal behaviors show that 1D frameworks with extended H-bonds, whether with or without a metal center, have the same stability. Inversely, despite the dimensionalities, the same metallic intermediate and final compounds are obtained for the two Rb^+^ ferroïc materials.

## 1. Introduction

In hybrid materials and from a crystal engineering point of view, the pioneering work of Yaghi and co-workers [1,2,3,4,5,6] has considerably enhanced the area of coordination polymers known as metal-organic frameworks (MOFs). In introducing the concept of secondary building units (SBUs) such as polycarboxylates, they have developed the understanding of supramolecular synthesis, where di or tetrameric carboxylate units on one hand, and H-bond networks on the other hand, play key roles in solid-state building. The intrinsic features of these two components govern the extended framework architecture through self-assembly process, involving among different interactions, short range and directional bonds.

In this context, and as part of our research concerning the polymeric metallic dicarboxylates, small and relatively rigid ligands stemming from oxalic acid have retained our attention, because this simplest dicarboxylic acid can give a wide variety of connectivity, thus generating a great number of oxalate-based metal structures [7,8]. 

Our interest in these materials is also driven by the wide range of applications using their electrical conductivity [9,10] and ferroïcity [11,12]. Such oxalate complexes are also developed for ion exchange [13] and catalysis [14]. Moreover, by their controlled thermal decomposition, they can be used as precursors in the preparation of advanced materials based on metal oxide nanoparticles. [15]. Within a systematic investigation concerning structure-properties relationships in dicarboxylate complexes [16,17,18,19,20,21,22,23,24,25,26,27], and especially metallic oxalates, we have focused on heterometallic connectors using alkali and transition metals, but we have obtained as by-products, also homonuclear compounds. 

In this paper, we report studies of three oxalato-bridged compounds: the two previously known Rb oxalates [28] and the novel H_3_O(HC_2_O_4_)(H_2_C_2_O_4_).2H_2_O. The latter, with its chemical formula, synthesis conditions and isostructural features within s-block oxalates, may enrich the controversial debate occurring in the pharmaceutical field about salts and cocrystals [29,30,31].

## 2. Experimental

### 2.1. Syntheses and Characterizations

All reagents were purchased from commercial sources and used without purification. Potentiometric titrations with potassium permanganate and sodium hydroxide, together with an Inductively Coupled Plasma Atomic Emission Spectroscopy analysis, on ICP-VARIAN, were carried out to determine oxalate, hydrogen contents, and the percent of rubidium, respectively. Infrared spectra were recorded in the 4,000–400 cm^-1^ range on a Perking-Elmer FTIR spectrophotometer, using the KBr pellet technique.

Thermogravimetric and differential thermal analyses (TGA, DTA) were conducted on a LABASYS-SETARAM instrument at heating rate of 10 °C min^-1^ in a Pt crucible, from ambient to 800 °C, under argon (**1** and **3**) and under helium (**2**). The dielectric permittivity of **3** was measured under dry argon, as a function of both temperature (100–500 K) and frequency (10^2^–10^5^ Hz), using a RLC bridge. 

The thermoelectric power was measured from room temperature to 500 K, under dry argon, by the differential technique using a digital micro voltmeter (Tacussel, Aris 2000). Prior to these measurements, the powdered samples were uniaxially pressed into disks of 8mm diameter and 1mm thickness. An electrical contact was obtained by cathodic sputtering.

#### 2.1.1. {Rb(HC_2_O_4_)(H_2_C_2_O_4_)(H_2_O) _2_}_∞_^1^

This compound (**1**) was obtained from a mixture of rubidium carbonate, aluminium hydroxide and oxalic acid dihydrate in a 3:1:3 ratio. The amounts of reactants were introduced into a 23 mL Teflon-lined stainless steel vessel. The vessel was sealed and heated at 120 °C for 24 hours. After cooling to room-temperature, single crystals were separated by filtration. Chemical analysis %: Calcd: Rb 28.44; C_2_O_4_^2-^ 58.57; H 0.99. Found: Rb 28.06; C_2_O_4_^2-^ 58.39 ; H 0.95 . IR data(cm^-1^): 3,490 (vs), 3,400–3,200(s), 1,690(s), 1,470(vw), 1,330 and 1,400(vw), 1250(s), 1,120 and 1,090(m), 850 and 775(vw), 720(s), 600(m), 570(m), 480(m). The thermogram undergoes four distinct steps, and several endothermic peaks, the largest one between 120 and 180 °C and others at 210, 260 and 550 °C, all connected with weight losses. Moreover, another three endothermic peaks, revealed at 380, 388 and 423 °C, occur without any weight loss, indicating solid-state transformations. Under a polarizing microscope, the single crystals showed domains that may be of ferroïc kind, as found in compound (**3**).

#### 2.1.2. {H_3_O(HC_2_O_4_)(H_2_C_2_O_4_).2H_2_O}_∞_^1^

Single crystals of this compound (**2**) were grown from a mixture of caesium chloride, chromium hydroxide and oxalic acid dihydrate under the same conditions. Chemical analysis %: Calcd: C_2_O_4_^2-^ 75.21; H 1.75. Found: C_2_O_4_^2-^ 74.98; H 1.80. IR data(cm^-1^): 3,500(s), 3,400–3,100(vs), 3,000(s), 1,690(m), 1,400(s), 1,270(m), 1,120(s), 850 (w) and 780(w), 710(s), 570(w), 505(m) 480(m). TGA curve shows two discrete losses; one followed by a narrow pseudo-plateau, and the second spread over a broad temperature range. 

#### 2.1.3. {Rb(HC_2_O_4_)}_∞_^3^

Single crystals were grown by a conventional synthetic route, using rubidium carbonate (0.231 g; 1 mmol.), oxalic acid (0.126 g; 1 mmol.), and chromium oxide (0.304 g; 2 mmol.), dissolved in deionized water. After addition of methanol, colourless crystals appeared in mother-liquor, after one week, under ambient conditions. Chemical analysis %: Calcd: Rb 48.98; C_2_O_4_^2-^ 50.43; H 0.57. Found: Rb 49.41; C_2_O_4_^2-^ 49.88; H 0.59 . IR data (cm^-1^) : 3,700–3,150(vs), doublet: 1,720 and 1,707(s), 1,630(s), 1,470(w) 1,410(s), 1,317(vw) and 1,286(s), 1,220(s) and 1,110(s), 870 and 850 (m), 720(vs), 590(w), 500(s), 475(s), 420(w). The TG/DTA analyses show two distinct steps displaying strong endothermic peaks; the largest one between 250 and 280 °C, and a second at 550 °C. Moreover, four weaker endothermic peaks, revealed at approximately 70, 175, 380 and 500 °C, occur without any weight losses, indicating solid-state transformations. Ferroïc domains on single crystals, observed under a polarizing microscope, are confirmed by thermal variation of the real part of relative permittivity ε’_r_, which displayed several peaks before the decomposition at 500 K: one at 310 K and successive peaks in the 390–480 K region.

### 2.2. X-Ray Crystallography

X-ray powder diffraction (XRPD) spectra of **1** and **3** were collected on a Phillips diffractometer with CuKα radiation in the range 5°< 2θ < 80° with a step of 0.02° (θ) and an acquisition time of 2 s per step. They were different from those of the starting materials and they confirm the purity of the bulk sample. The moisture of the powdered compound **2** did not allow the XRPD spectrum to be obtained. However, the simulated ones showed that **1** and **2** are isostructural.

The reflection intensities were collected on a Bruker-Nonius Kappa CCD diffractometer at 298 K. Analytical absorption corrections [32] were applied. After data reduction using EVALCCD programs [33], the structures were solved by direct methods and subsequent Fourier analyses. In case of compound **3**, the unique hydrogen atom was located in difference-Fourier map and refined freely. Concerning the isostructural compounds, some H atoms were found in difference-Fourier map and the others placed in calculated positions. The three structures were refined on F^2^, by full-matrix least-squares, with anisotropic thermal parameters for all non-hydrogen atoms. SHELXS97 and SHELXL97 programs, under WinGX interface [34,35,36] were used for computations.

Crystal data and experimental details of data collection and refinement are summarized in Table 1. Selected geometric parameters are listed in Table 2. 

**Table 1:** Crystallographic data for **1**, **2** and **3**.

Compound	1	2	3
Crystal data			
Empirical formula	C_4_ H_7_ O_10_ Rb	C_4_ H_10_ O_11_	C_2_ H_1_ O_4_ Rb
Formula mass	300.57	234.12	174.50
Space group	P-1	P-1	P2_1_/c
a (Å)	6.2969(10)	6.3310(1)	4.2940(3)
b (Å)	7.206(3)	7.22649(10)	13.6229(2)
c (Å)	10.641(2)	10.5541(2)	7.6689(1)
α (°)	94.549(2)	94.253(2)	90.00
β (°)	100.3990(10)	100.2630(5)	101.50(5)
γ (°)	97.676(2)	97.719(2)	90.00
*V* (Å^3^)	468.0(2)	468.440(14)	439.60(9)
Z	2	2	4
*T* (K)	298(2)	298(2)	298(2)
*ρ*_cal_ (g.m^-3^)	2.133	1.660	2.637
*λ*(Mo K_α1_) (Å)	0.71070	0.71070	0.71070
*μ* (mm^-1^)	5.332	0.175	11.150
Data collection			
*θ*_max_ (°)	30	27.49	30
h	-8 ≤ h ≤ 8	-8 ≤ h ≤ 8	-6 ≤ h ≤ 6
k	-10 ≤ k ≤ 10	-9 ≤ k ≤ 9	-18 ≤ k ≤19
l	-14 ≤ l ≤ 14	-13 ≤ l ≤ 13	-10 ≤ l ≤ 10
Reflections collected/unique	17,670/2,712	15,199/2,143	9,372/1,278
Rint	0.1234	0.0466	0.1126
*T*_max_; *T*_min_	0.527; 0.289	0.968; 0.958	0.315; 0.111
Refinement			
Refinement method	Full-matrix	Full-matrix	Full-matrix
	least-squares on *F*^2^	least-squares on *F*^2^	least-squares on *F*^2^
Parameters	165	177	69
Goodness-of-fit on *F*^2^	1.160	1.056	1.123
Final R indices [*I*>2σ(*I*)]	R1 = 0.0351	R1 = 0.0458	R1 = 0.0317
	wR1 = 0.0834	wR1 = 0.1158	wR1 = 0.0690
R indices (all data)	R2 = 0.0396	R2 = 0.0670	R2 = 0.0433
	wR2 = 0.0854	wR2 = 0.1256	wR2 = 0.0744
∆*ρ* max;∆*ρ* min (eÅ^-3^)	1.626, -1.125	0.556, -0.468	0.803, -0.670

**Table 2:** Selected geometric parameters (Å, °) for **1**, **2** and **3**.

**Compound 1 ^a^**			
Rb-O41^i^	2.9554(18)	O2w-Rb-O42	54.09(6)
Rb-O21^ii^	2.9744(19)	O1w-Rb-O22^iv^	148.33(5)
Rb-O11	2.9845(19)	O11-C1-C2-O21	178.5(2)
Rb-O1w	3.025(2)	O11-C1-C2-O22	-1.6(3)
Rb-O2w	3.109(3)	O12-C1-C2-O21	-0.3(3)
Rb-O22^iv^	3.173(2)	O12-C1-C2-O22	179.5(2)
Rb-O42	3.238(2)	O31-C3-C3^v^-O32^v^	1.2(3)
Rb-O31	2.9719(19)	O41-C4-C4^iii^-O42^iii^	0.5(3)
Rb-O12^ii^	2.9700(2)		
**Compound 2**			
O3w-O41	2.939(2)	O11-C1-C2-O21	-178.9(2)
O3w-O31	2.950(2)	O11-C1-C2-O22	0.7(3)
O3w-O11	2.972(2)	O12-C1-C2-O21	0.3(3)
O3w-H5w	0.923(10)	O12-C1-C2-O22	179.95(19)
O3w-H6w	0.922(10)	H5w-O3w-H6w	107(3)
O3w-H7w	0.93(3)	H5w-O3w-H7w	121(3)
		H6w-O3w-H7w	104(3)
**Compound 3 ^b^**			
Rb-O2^v^	2.839(2)	Rb^iv^-O3-H	127(3)
Rb-O4^i^	2.908(3)	Rb^vi^-O3-H	73(3)
Rb-O1^ii^	2.940(2)	O1-C1-C2-O3	164.8(2)
Rb-O1	2.945(3)	O1-C1-C2-O4	-14.8(4)
Rb-O4^ii^	2.957(3)	O2-C1-C2-O3	-15.8(4)
Rb-O1^iii^	2.980(3)	O2-C1-C2-O4	164.5(2)
Rb-O3^v^	3.027(2)		

^a^ Symmetry transformations used to generate equivalent atoms: (i) x-1, y, z; (ii) x, 1+y, z; (iii) 1-x, -y, 1-z; (iv) 1-x, -y, 2-z; (v) 1-x, 1-y, 1-z. ^b^ Symmetry transformations used to generate equivalent atoms: (i) 1+x, y, 1+z; (ii) -x, 1-y, -z; (iii) 1-x, 1-y, -z (iv) x, 3/2-y, z-1/2; (v) x, 3/2-y, ½+z; (vi) 1-x, 3/2-y, z-1/2.

## 3. Results and Discussions

### 3.1. Syntheses and Characterizations

Single crystals of these three oxalate-based homonuclear materials have been isolated as by-products. Although it was generally found that an increase in reaction temperature favors anhydrous compounds formation, the two isostructural crystals **1** and **2**, obtained hydrothermally, contain water molecules, whereas **3** resulting from soft chemical route at room temperature is completely anhydrous. Moreover, the partially deprotonated ligand appeared to depend roughly on the metal ligand ratio and probably on reaction time [18,20,25,37]. As shown in a comparative study in the series of α,ω-aliphatic dicarboxylic acids [16,17,18,19,20,21,22,23,24,25,26,37], the optimal hydrogen bonding patterns should be more determining in the deprotonation of these acids than their corresponding pKa. The nature of alkali salts used as starting materials has probably a great effect on the formation of this kind of material, through the pH value of the mixture [38,39,40,41,42,43,44]. This factor seems to be also important in the synthesis of similar materials used for therapeutic purpose, and described as multi-component systems, although the synthetic routes commonly carried out in this case are soft and use only organic compounds such as amines and acids as starting materials [30,31].The unit-cell parameters and the peak intensities obtained from XRPD for compounds 1 and 3, are in agreement with those derived from single crystal diffraction. On the basis of previous IR studies dealing with metallic carboxylate and oxalate compounds, the nature and coordination of oxalate groups and water molecules have been deduced [45,46,47,48,49,50,51,52,53,54,55,56]. 

The IR spectra of **1** and **2** show some similarities, essentially concerning strong hydrogen bonds and water molecules. The broad bands in the 3,680–3,100 cm^-1^ region correspond to stretching vibrations ν_s_(O-H) and ν_as_(O-H) of water molecules. The characteristics of these latter are also inferred from the presence of bands at 720 and 570 cm^-1^ corresponding to rocking vibration **ρ**_r_ (H_2_O) and wagging vibration **ρ**_w_(H_2_O). Moreover, the broad band in the range 3,300–3,100 cm^-1^, observed also in the oxalic acid dihydrate, can be assigned to strong hydrogen bonds. Concerning the oxalate groups, the vibration frequencies appearing between 1,700 and 1,000 cm^-1^ confirm their various coordination modes *via* one to four oxygen atoms. The two spectra give also characteristic ν_as_(C-O), ν_s_(C-O), ν_s_(C-O)+ **δ**(COO) vibrations of oxalate ligands, localized at 1,690, 1,470, 1,400, 1,250 cm^-1^ for **1**, and at 1,690, 1,400, 1,250 cm^-1^ for **2**, indicating the presence of chelating unidentate or bridging polydentate ligands. It is interesting to point out that the major differences between the two spectra come mainly from the effect of hydronium ion and the whole hydrogen bond network. Thus, the weak bands observed in the region 1,500–1,300 cm^-1^ for **1**, like in oxalic acid dihydrate, are replaced in **2** by a sharp and strong band at 1,400 cm^-1^, which can be attributed to ν_s_(C-O) [45,46,47,48,49,50,51,52,53,54,55,56]. This difference suggests a more ionic lattice for hydronium compounds. Besides this, as expected the peaks at 1,120 and 1,090 cm^-1^ in **1** correspond to free OH groups and bonded OH groups, respectively, while only one peak is obtained at 1,120 cm^-1^ in **2**, indicating free OH groups. Compound **1** exhibits a band at 590 cm^-1^, due to Rb-O bonds. Compound **2** can also be distinguished by an additional strong band in the range 3,000–2,700 cm^-1^ with two shoulders observed at 3,000 and 2,750 cm^-1^. 

According to recent investigations focused on hydronium ion [57], where it appears that the band positions are not sensitive to anion nature nor to the counter ion, these peculiar bands may represent the overlapped ν_s_(O-H)_3_ and ν_as_(O-H)_3_ stretching vibrations. Moreover, the band due to bending vibration **δ**_as_(HOH)_3_, observed in the investigated monohydrate acids in the range 1,550–1,650 cm^-1^ [57], is here overlapped with the strong band due to the carboxylate function localized precisely at 1,690 cm^-1^.

The IR spectrum of **3** shows two important regions in the frequency range 3,700–3,080 cm^-1^ and 1,730–400 cm^-1^. In the high frequency range, the broad band centered at 3,500 cm^-1^ can be assigned to H-bonded hydrogen oxalate groups vibrations*.* More interesting are the peaks in the region 1,730–1,000 cm^-1^. The bands at 1,720 and 1,710 cm^-1^ are attributed to C=O stretching of COOH group and the peaks at 1,630 [νas(C-O)], 1,470 and 1,410 as well as those at 1,286 and 1,220 [νs(C-O)], 1,110 [δ(C-OH] and 722 cm^-1^ [δ(OCO] support the presence of bridging-chelating oxalate group [45,46,47,48,49,50,51,52,53,54,55,56]. Moreover, the band observed near 590 cm^-1^, which is, as expected, absent in compound **2**, is characteristic of Rb-O stretching.

### 3.2. Thermal and Electric Behaviors 

#### 3.2.1. {Rb (HC_2_O_4_) (H_2_C_2_O_4_) (H_2_O)_2_}_∞_^1^

The total weight losses, and the white powder obtained, are in good agreement with achievement of Rb_2_CO_3_ residue (weight losses: calcd: 61.56%; found: 60.72%). Four stages appear in the thermogram and several endothermic effects are detected on DTA curve. The first one, associated with departure of the two water molecules (calcd: 11.98%; found: 11.40%), occurs between 120 and 180 °C. The strong and large corresponding peak may be due to the formation of the anhydrous Rb(HC_2_O_4_).H_2_C_2_O_4_ as an intermediate compound, which is stable in a narrow temperature range. As brought out further from X-ray determination, only one O atom of each independent oxalic acid belongs to the first coordination sphere of the metal, while the ionized HL ligand contributes three out of four O atoms. Moreover, one of the two independent oxalic acid molecules is tightly held to one aqua ligand (O42–H42….O1w) and is not connected to HL *via* H-bond as does the second oxalic acid molecule (O32–H32….O22). Therefore, the simultaneous release from the metal coordination sphere of one acid molecule in the chemical formula, without collapse of the structure, is quite possible. This proposal can be reasonably assumed, corroborated by a similar behavior, in Ba(C_2_O_4_)(H_2_C_2_O_4_).2H_2_O, where the departure of oxalic acid is associated with single crystal formation, on the crucible faces, of BaC_2_O_4_ [58]. 

Two consecutive steps giving rise to sharp endothermic peaks take place between 180 and 250 °C, corresponding to oxalic acid decomposition into CO, CO_2_, and H_2_O (weight losses: calcd: 30.56%; found: 30.45%). The relatively high temperature of this event indicates that the crystallinity is retained, unlike what it is noticed in dehydration of commercial H_2_C_2_O_4_.2H_2_O, which starts from 40 °C [59]. At 260 °C, the rubidium hydrogen oxalate transforms to rubidium oxalate, stable in a broad temperature range up to 550 °C (weight loss: calcd: 14%; found: 14.30%). The peak at 550 °C, detected in DTA curve, associated with 4.70% weight loss (calcd: 4.61%), is consistent with the formation of Rb_2_CO_3_ and generation of CO. The results obtained indicate that the thermal behavior of this rubidium compound proceeds as follows:

Rb(HC_2_O_4_)(H_2_C_2_O_4_)(H_2_O)_2_ ⟶ Rb(HC_2_O_4_).H_2_C_2_O_4_ + 2H_2_O

Rb(HC_2_O_4_).H_2_C_2_O_4_ ⟶ Rb(HC_2_O_4_) + CO_2_ + CO + H_2_O

Rb(HC_2_O_4_) ⟶ 1/2 Rb_2_C_2_O_4_ + CO_2_ + 1/2 H_2_

1/2 Rb_2_C_2_O_4_ ⟶ 1/2 Rb_2_CO_3_ + 1/2CO

Before the decomposition stage into rubidium carbonate, the DTA curve reveals three endothermic peaks, localized at 381, 388 and 423 °C, without any weight loss, indicating the solid state transformations within the four Rb_2_C_2_O_4_ polymorphs [60,61,62]. Our results agree globally with those reported for the three phase transitions and the narrow temperature domain stability of the monoclinic form [62]. However, the transition temperatures found here, even lying in the onset values, are slightly different. This is not surprising since the freshly prepared rubidium oxalate used by Dinnebier *et al.* [62], consists of both δ and γ polymorphs. Besides, it is well known that the occurrence of phase transition depends upon the nature of starting materials as well as the heating rate. Finally, the latest weaker peak observed at 640 °C on heating corresponds to phase transition of Rb_2_CO_3,_ in agreement with literature data [62].

#### 3.2.2. {H_3_O(HC_2_O_4_)(H_2_C_2_O_4_).2H_2_O}_∞_^1^

There is only one stage of dehydration, between 120 and 180 °C, followed by a narrow pseudo-plateau until 220 °C (weight losses: calcd: 15.32% ; found: 15.40%). The decarboxylation pathways proceed *via* two indiscernible stages spread over about a hundred degrees (until 300 °C), associated with 84% mass loss (calcd: 84.60%), which is attributed to the release of CO_2_, CO and H_2_O. This brings a total loss of the original sample mass without any residue, like in a reported oxalic acid, even though it is generated differently [59]. Therefore, we propose this thermal behavior scheme:

H_3_O(HC_2_O_4_)(H_2_C_2_O_4_).2 H_2_O ⟶ H_3_O(HC_2_O_4_)(H_2_C_2_O_4_) + 2H_2_O

H_3_O(HC_2_O_4_)(H_2_C_2_O_4_) ⟶ 2CO_2_ + 2CO + 3H_2_O

#### 3.2.3. {Rb(HC_2_O_4_)}_∞_^3^

This anhydrous complex is stable up to 200 °C. The observed (34.75%) and calculated (33.81%) total weight losses are in good agreement with achievement of carbonate salt residue at about 800 °C, as expected [62]. The TG curve of RbHC_2_O_4_ shows clearly two well-defined steps. The first one occurs at 220–280 °C, accompanied with two successive and strong peaks in the DTA curve. According to the observed weight loss of 25.92% (calcd: 25.78%), the release of one CO_2_ molecule and one half H_2_ molecule is proposed, corresponding to the formation of Rb_2_C_2_O_4_, this later being stable up to about 550 °C. The second stage takes place at 520–550 °C, and can be attributed to the formation of Rb_2_CO_3_ with generation of CO, as indicated by the mass loss of 7.85% (calcd: 8.02%).This transformation from oxalate to carbonate is usually observed in alkali metal oxalate at the same temperature [62,63]. According to these results, the proposed thermal behavior is:

Rb(HC_2_O_4_) ⟶ 1/2 Rb_2_C_2_O_4_ + CO_2_ + 1/2 H_2_

1/2Rb_2_C_2_O_4_⟶ 1/2Rb_2_CO_3_ + 1/2 CO

Moreover, several endothermic peaks appear in the DTA curve, without any weight loss. In the temperature range stability of Rb_2_C_2_O_4_, two weak signals, occurring at 380 °C and 500 °C, can be related to γ → β→ α phase transitions of the anhydrous rubidium oxalate, as previously reported [61,62,63]. Furthermore, and before the decomposition stage, two more important peaks appear at approximately 70° and 175 °C, and this may be due to some change in the structure and ferroïc properties of the investigated complex.

**Figure 1:**
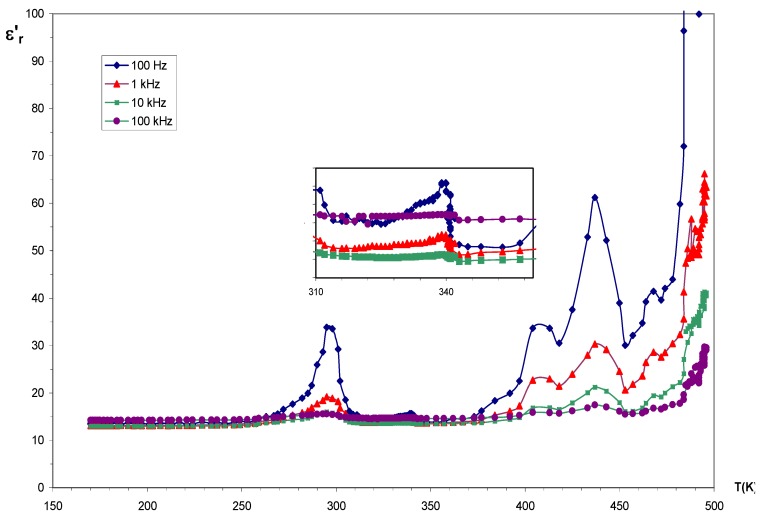
Thermal evolution of the permittivity ε’_r_ for compound **3**.

Indeed, as it appears in Figure 1, which shows the thermal evolution of ε’_r_, a weak maximum at 340 K, and in the successive peaks revealed between 390 and 480 K, the maximum localized at 440 K correspond to the phase transition detected in DTA curve.In the same way, thermal dependence of the conductivity shows frequency dispersion and insulator character. At low temperature (170 < T < 290 K), as expected, the weak conductivity remains constant, mainly at low frequency (σ~10^-11^ Ω.cm^-1^). From 390 to 460 K the conductivity increases, and its behavior in the higher temperature phase is consistent with Arrhenius law. Most important are the same anomalies observed in these two curves, particularly in 390–460 K region.

On the basis of our structural results, we can relate the phenomenon occurring in this temperature range to the superposition of several mechanisms such as the breaking of H bonds, the probable rotation of the twisted oxalate groups during the phase transformation, and the beginning of the decomposition. The phase transformation can be considered as structural, owing to the fact that in the Seebeck coefficient plot, S = f(T), a discontinuity at approximately 335 K and a broad peak at 400 K are observed, indicating a possible gradual change in the structure. The S values are positive and imply a cationic conduction, probably due to proton motion.

Besides these experimental results, it is noteworthy from the literature that several oxalate materials are distinguishing by common structure-thermal behavior-property relationships, implying peculiar H-bonds and succesive phase transitions of ferroïc and/or superionic nature [10,11,39]. Therefore, and supported by the anisotropic strain found previously in this compound [44], and by all thermal, structural, and electric results we have obtained on this crystal, we can suggest that the first phase transition is of ferroïc nature, and the second one is probably due to H motion.

### 3.3. Crystal Structures

Crystal structures reveal that the two isostructural compounds **1** and **2** are closely related to the family of dihydrates known as tetroxalates [38,39,40,41,42,43] or trihydrogen oxalate [28,44], comprising of metals, ammonium, potassium, caesium, thallium and rubidium analogs [28,38,39,40,41,42,43,44,64]. Formulated MH_3_(C_2_O_4_)_2_.2H_2_O or M(HC_2_O_4_)(H_2_C_2_O_4_).2H_2_O are considered as superacid salts. The Rb compound has been already described [28]. However, its structure reinvestigation using supplementary and more accurate data, allows the localizsation of all H atoms, which bring out its 1D polymeric structure that can be better affiliated to coordination polymers. This complex is the poly[diaqua(hydrogenoxalato) (dihydrogenoxalato) rubidium(I)]: Rb(HC_2_O_4_)(H_2_C_2_O_4_)(H_2_O)_2 ._

 The hydronium compound **2** is new in the s-block series. In spite of its isostructural characteristics with compound **1**, it corresponds to H_3_O(HC_2_O_4_)(H_2_C_2_O_4_).2H_2_O formula and represents the poly[(hydrogenoxalato)(dihydrogenoxalato) hydronium dihydrate]. It resembles, at first sight, one dimorphic oxalic acid recently described as sesquihydrate [29]. Here, its structural study shows that, although its chemical formula fits well with the stoichiometry [1H_2_C_2_O_4_][3(1/2 H_2_O)], and the molecular moieties indicated in the dimorph reported, there are some discrepancies between the two crystallography investigations, the supramolecular characteristics, and the resulting explanations concerning the nature of interactions that bind the components into crystals. 

#### 3.3.1. {Rb(HC_2_O_4_)(H_2_C_2_O_4_)(H_2_O)_2_}_∞_^1^

The asymmetric unit contains one Rb cation, one partially deprotonated ligand (HL), two half oxalic acid molecules (H_2_L), and two water molecules.

The framework is built from isolated RbO_7_(H_2_O)_2_ polyhedra, bridged in [011] direction by two carboxylate ligands (HL and one H_2_L). The third ligand connects the polyhedra in roughly [001] direction. In this polymeric packing, the (001) plane is the most dense, and the framework stability is maintained by an extensive hydrogen bond network involving all O atoms of hydrogen oxalate anion, unionized oxalic acid molecules and water molecules.

**Figure 2:**
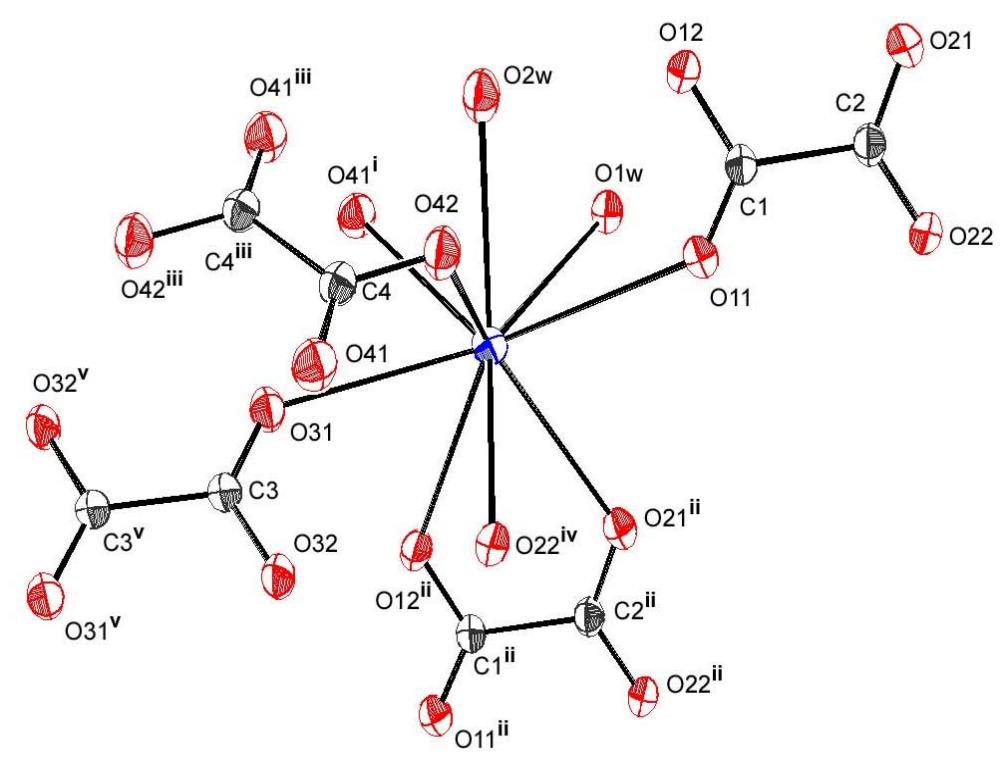
Ortep view showing asymmetric unit and Rb environnement in compound **1**. Displacement ellipsoids are drawn with 50% probability.

As seen in Figure 2, the first coordination sphere of Rb atom is made of five oxygen atoms from two HL and two H2L, completed by oxygen atoms from independent aqua ligands. In the second coordination sphere, two contacts > 3.10Å are provided by one HL (O22) and one H2L (O42). The three independent ligands are in a rather usual *anti-anti* conformation (Table 2). The corresponding coordination polyhedron is distorted as a consequence of the bite angles, ranging from 54.13(6)° to 148.34(5)°, and can be described as a monocapped tetragonal antiprism (O12, O22, O11, O1w and O31, O41, O42, O2w being the two approximately parallel square planes), with the axial cap localized on O21. The Rb-O bond lengths vary between 2.953(5) and 3.236(4) Å (Table 2), and are consistent with expected values [28,62,65]. The ionized ligand involves all its O atoms, including the protonated one, giving a chelating malonate mode. Despite the fact that in this ligand the two end functional groups are different, if we take into account the second coordination sphere, the same coordination mode distinguishes them, bringing out μ-_1,3_ bridges. Consequently, HL is surrounded by three cations, and is in µ_4_ coordination mode, containing once more, η^4^ chelation. 

Symmetry codes : (i) x-1, y, z; (ii) x, 1+y, z; (iii) 1-x, -y, 1-z; (iv) 1-x, -y, 2-z; (v) 1-x, 1-y, 1-z.

Concerning the two unionized ligands, one involves each end *via* the single deprotonated oxygen, in bis-unidentate fashion, and is surrounded by two metals, while the second is bis-bidentate, involving all its oxygen atoms in two µ_1,3_ bridges, if we consider the relatively long Rb-O42 bond. Overall, it is, in this case, in µ_4_ coordination mode, surrounded by four cations. The hydrogen bonding pattern shows strong and linear bonds, where the two oxalic acid molecules, as well as the single anion, act as donors involving, each of the three, one protonated oxygen atom in the shortest hydrogen bonds, between 2.489(3) and 2.542(3)Å. The moderate hydrogen bonds concern one water molecule (O1w) as donor, the acceptor being the ionized ligand, which involves the two deprotonated oxygen atoms not implied in the precedent strongest H-bonds. The second water molecule (O2w) is in the centre of four O2w…O contacts, where the two independent H_2_L ligands act as acceptors in the two resulting bifurcated hydrogen bonds [66]. Within this scheme, the minor component for one acid molecule concerns O32 atom. One restraint applied in the final refinement corresponds precisely to O32-H32 bond where H32 appears to have a relatively high U_eq_ (0.11(2) Å^2^). For the second acid molecule, it is the major component implying O42 which seems to have an effect upon short D-H …. H-D *via* H42 atom attached to it. Others restraints have been used for refining H atoms of water molecules in order to reduce the discrepancies between O-H distances within water molecules. However, another short contact is observed between one H atom of the second water molecule (H3w) and the end functional group of the ionized ligand (H12).

#### 3.3.2. {H_3_O(HC_2_O_4_)(H_2_C_2_O_4_).2H_2_O}_∞_^1^

**Figure 3:**
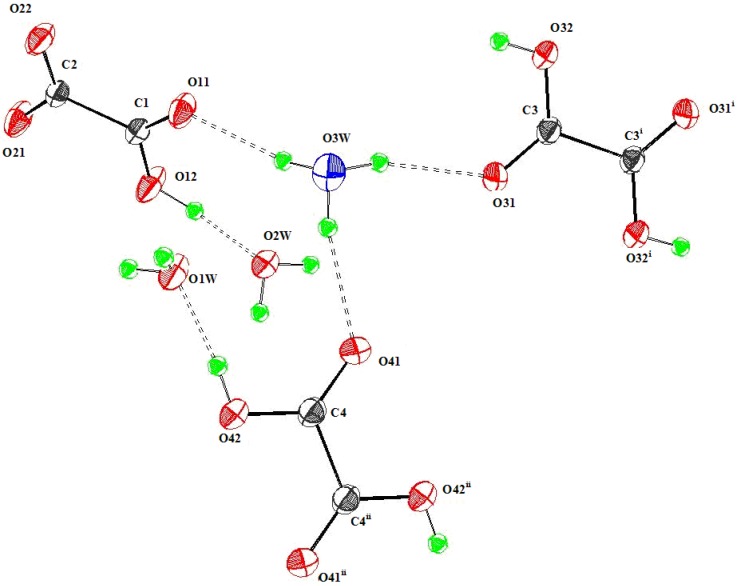
Molecular view of compound **2**. Displacement ellipsoids are drawn with 50% probability.

The asymmetric unit contains one H_3_O^+^ cation, one HL, two water molecules and two half H_2_L. Whereas in the sesquihydrate reported [29], four positions have partial occupancy, here all occupancy factors are in good agreement with crystallographic site multiplicity. The same network than 1, in which H_3_O^+^ replaces Rb^+^, exhibits a very different binding situation, owing to the symmetry and nature of the hydronium cation (Figure 3).

In fact, H_3_O^+^…..O interactions occur *via* H-bonds and are longer (between 2.939(2) and 2.972(2) Å) than other H-bonds observed in the all hydrogen-bonding pattern (between 2.470(2) and 2.925(2) Å) Within H_3_O^+^, the three equivalent O-H bonds are somewhat longer (0.92(2) Å) than those formed in each of the two water molecules (0.84(2) to 0.85(3) Å).

In the extensive hydrogen bond scheme, the shortest bonds involve, as donors, the three protonated independent O atoms in strong linear directionality, the acceptors being the two water molecules and the deprotonated atom of HL. H_3_O^+^ acts as donor in moderate electrostatic H-bonds with 1HL and 2 H_2_L *via* their deprotonated O atoms which are in turn implied, always as acceptors, and in moderate bonds, with O1w (O1w – H11 …..O11) and O2w (O2w– H3w...O31 ; O2w –H4w…O41) [66].But H_3_O^+^ is not attached to water molecules, unless we accept the longest interactions equal to 3.058(2) and 3.187(2)Å. Moreover, its geometry is approximately a trigonal pyramid with a nearly C_3v_ symmetry (Table 2). We can obviously consider that no Zundel cation H_5_O_2_^2+^ (known for its D_2h_ symmetry), nor H_7_O_3_^+^ units, are formed in this framework, despite the presence in it of two water molecules [67]. Consequently, the resulting structural characteristics of this new compound show that it may be placed in the so-called solid state acid dihydrates [H_3_O^+^.3B], where B is a proton acceptor conjugate base anion (here HC_2_O_4_^-^) and neutral molecule (here H_2_C_2_O_4_) [57]. For example, it exhibits some common features with barium oxalate acid dihydrate [58] and caesium tetroxalate dihydrate [43]. Futhermore, owing to its isostructural characteristics with compound **1**, it is more closely related to coordination polymers than cocrystals or salts. However, unlike compound **1**, and ammonium or potassium analogous compounds, a bifurcated hydrogen bond is formed around one of the two water molecules, giving one three-centred hydrogen bond [66]. Moreover, water molecules act also as donors with one HL (O1w) and the two H_2_L (O2w).

Symmetry codes : (i) 1-x, -y, 2 -z; (ii) 3-x, 1-y, 2-z.

**Figure 4:**
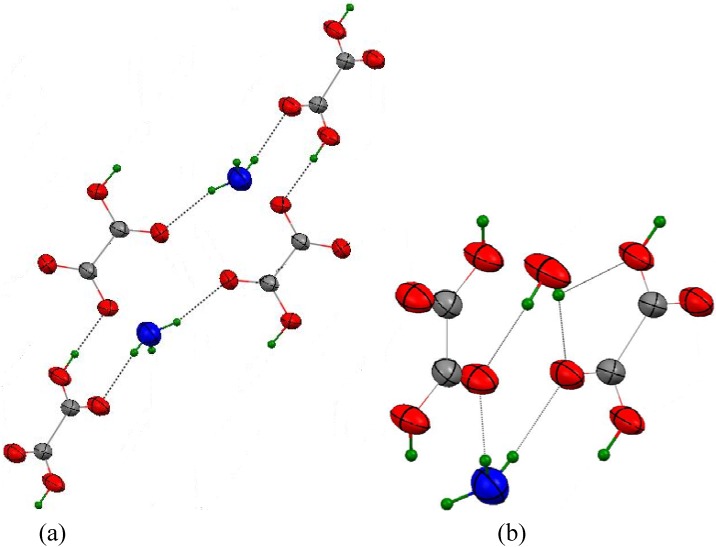
(a)The centrosymmetrical hydrogen-bonded motif, with graph notation R_6_^6^(22), (b)The homosynthon, with graph notation R_4_^2^(8) in compound **2**.

In spite of its isostructural characteristics with compound **1**, the hydronium compound corresponds to H_3_O(HC_2_O_4_)(H_2_C_2_O_4_).2H_2_O formula and represents the (hydrogenoxalato) (dihydrogenoxalato) hydronium dihydrate. From a chemistry point of view, it is different from oxalic acid dihydrate in its α or β forms based on neutral species [68,69]. From a structure point of view, it is also different from one dimorphic sesquihydrate of oxalic acid [29]. Although this latter has been structurally studied at a lower temperature (273 K), a statistic disorder affects H_3_O^+^ leading to unsatisfactory chemical formula sum, written C_4_H_9_O_11_, and one wrong R_4_^2^(8) synthon implying two ionized ligands, as acceptors. The structure obtained here, without any kind of positional disorder, allows the recognition of supramolecular dimensionality based on bifurcated hydrogen bonds. Within this pattern of simple and bifurcated hydrogen bonds, several kinds of hydrogen-bonded motifs, are responsible for the overall organization [70,71].Moreover, the unique R_4_^2^(8) synthon is unsymmetrical and involves two neutral ligands as acceptors, and 1 H_2_O and 1 H_3_O^+^ as donors. Figure 4 shows the unique centrosymmetrical R_6_^6^(22) hydrogen-bonded motif, and the unique R_4_^2^(8) homosynthon, which is unsymmetrical. All these results invalidate also theoretical predictions giving an ionized solid containing C_2_O_4_^2-^ and 2 H_3_O^+^ [72]. 

Scientific literature surveys indicate that in structure determination, a hydronium cation is proposed essentially in order to provide charge balance and it is often disordered, unlike water molecules [29,73,74]. Moreover, it can play the role of ligand, counterion or lattice ion in between the layers [73,74,75,76]. Here, the isostructural character with Rb compound points out clearly that H_3_O^+^ may be considered as a connector in the resulting polymeric framework. This architecture, without any metal, but where H_3_O^+^ takes Rb^+^ place, calls into question the structural frontiers between superacid salts, cocrystals, and coordination polymers in supramolecular chemistry [77,78,79].

#### 3.3.3. {Rb(HC_2_O_4_)}_∞_^3^

**Figure 5:**
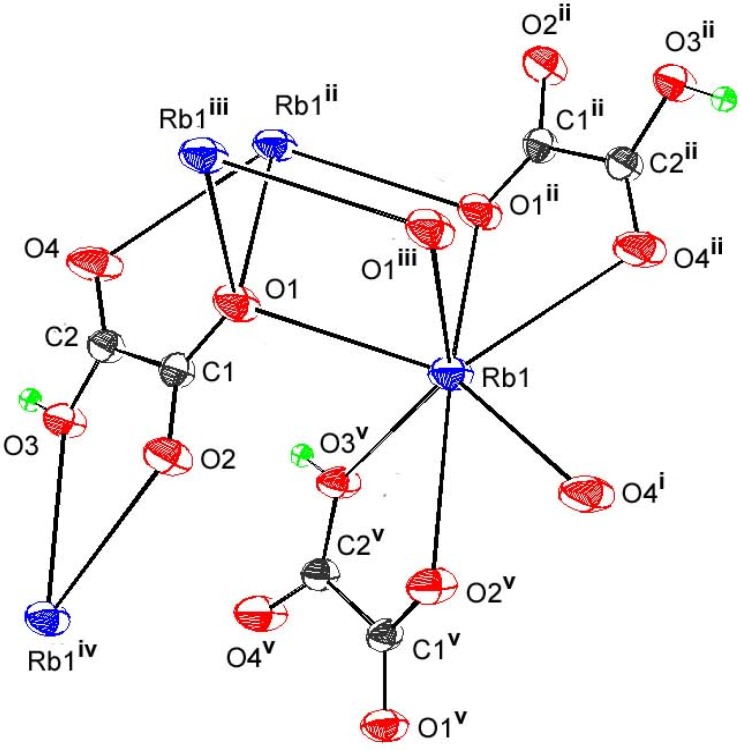
Ortep view showing cage-like assembly of compound **3**. Displacement ellipsoids are drawn with 50% probability.

In the infinite polymeric network, the three-dimensional layered dense framework is built up from cross-linked infinite chains of RbO_7_ polyhedra. The tritopic ligand is tetradentate, exhibiting two different η^4^ chelations and displaying one four-bonded oxygen atom forming cage-like assembly, as it appears in Figure 5. 

As mentioned in previous studies, this compound is isostuctural with the potassium analogous [80,81,82]: Rb atom is seven-coordinated by end functional O atoms from two chelating and three monodentate ligands. The ligand exhibits two η^4^ chelations known as "malonate" mode, one of them being bridging-chelating, involving O4 and O1. All the oxygen atoms of the ligand present four different links. By binding to three metals, O1 creates a cage-like network which is found when its donor character is enhanced as in "serendipitous assembly" [83]. O4 is triply ligating and acts as a bridge between two metals. The protonated oxygen O3 binds also to three atoms at all, but without bridging, and O2 is simply doubly ligating. As a result, the ligand is not planar but twisted about its C-C bond, the calculated dihedral angle between the two chelates, is 9.50(18)°.Within the ligand, other geometric parameters lie in the expected ranges [84], as well as the C-C bond which is somewhat longer like in many oxalate compounds [85,86].

The coordination polyhedron is a slightly distorted monocapped trigonal antiprism, with atom O4 in axial capping position. The Rb-O bond lengths (Table 2) are consistent with expected values [62,65]. The resulting framework is a dense three-dimensional layered structure, formed by cross-linked infinite chains of polyhedra sharing opposite edges, running along c and a axes. The corrugated resulting layers, parallel to (010), interconnected through the carbon backbone of the ligands, and *via* strong intermolecular hydrogen bonds H1...O2 (1.58(5)Å), lead to the formation of small rhombic voids in [010] direction. The reinvestigation of this compound, previously reported [82], gives supplementary and more accurate data, and confirms the geometric similarity with KHC_2_O_4_ and NH_4_(HC_2_O_4_) [80]. Moreover, present results bring out the twisted conformation of the ligand, the 3D polymeric network, and the leading part, taken in the packing, by H-bond pattern. 

Symmetry codes : (i) 1+x, y, 1+z; (ii) -x, 1-y, -z; (iii): 1-x, 1-y,-z; (iv): x, 3/2 -y, z-1/2; (v) x, 3/2 -y, 1/2+z.

## 4. Conclusion

During an attempt to get heteronuclear dicarboxylates, by using, beside transition metals, s-block elements as connector, (for exploiting their oxophilitic character), and oxalate as linker (for its dual-properties including those of aliphatic ligands and unsaturated ones, displaying relative rigidity and multiple bond interactions), we have obtained homonuclear polymeric compounds as by-products. 

The structural reinvestigation of the two rubidium-based hybrid materials, {Rb(HC_2_O_4_)(H_2_C_2_O_4_)(H_2_O)_2_}_∞_^1^ and {Rb(HC_2_O_4_)} _∞_^3^ brings out their self-assembly with different dimensionalities, their H-bond patterns forming zig-zag chains, or extensive 3D packing including bifurcated bonds, and their good thermal stability. The general structural trends in oxalate series, showing that high structural dimensionality corresponds to low hydration degree is confirmed, whereas, the generally admitted proposal assuming that, under hydrothermal conditions, there is trend towards obtaining MOFs with a lower void space and lower water content, is invalidated, the anhydrous compound being obtained by a soft chemical process, unlike the two isostructural aqua or hydrated compounds. In the ferroïc domain structure of hybrid materials, several solid state phase transitions, matching up with preliminary dielectric measurements, imply interesting non-linear properties. We have improved the structure-properties relationships on one hybrid materials, by performing some electric measurements.The ferroïc and non-linear properties displayed are very interesting and encourage us to add other studies and extend them to all oxalate hybrid compounds synthesized in our Laboratory.

The non-metallic-based crystal of {H_3_O(HC_2_O_4_)(H_2_C_2_O_4_).2H_2_O}_∞_^1^, presents un precedent affiliation to coordination polymers, by its architecture, its supramolecular interactions and its hydrothermally growth. Its thermal behavior, evidencing the same stability than the Rb compound, is another argument for considering this new compound as belonging to coordination polymer. Different hydrogen- bonded motifs generated by water molecules and hydrogenoxalate anion or by H_3_O^+^ and the neutral dihydrogenoxalate, related it to cocrystals. But, owing to the fact that all the starting materials were used in solid state, and that the hydronium complex formulation indicates a proton transfer, it can be placed also among the solid state super acid salts. On the basis of our IR data and single crystal structure, and taking into account the synthesis conditions, we can asses that the correct proton state formulation including an H_3_O^+^ with nearly C_3V_ symmetry without any disorder illustrates, in paraphrasing Childs *et al.* [31], a novel cocrystal-coordination polymer continuum. 

Therefore, the controversial debate occurring in the pharmaceutical field surrounding salts and cocrystals, tends to revive with the recent emergence of the concept of supramolecular medicinal chemistry, which uses the approach introduced by O.M. Yaghi and co-authors [1,2,3,4,5,6], in MOF’s. Illustrating this by the hydronium polymer, these results show that we may reasonably suggest, in the light of supramolecular chemistry, that the H_3_O^+^ polymeric compound is on the borderline of crystalline acid salts/ cocrystals/ coordination polymers. Its exact formula is analogous to NH_4_^+^ and Rb^+^ compounds, but with two lattice water molecules instead of two coordinated ones. With this example among s-block, it appears that H_3_O^+^ can play the same role than NH_4_^+^, and therefore may display the same non-linear properties.

## Supplementary Materials

Crystallographic data for the structural analysis of the two compounds have been deposited in the Cambridge Crystallographic Data Center CCDC N° 732601, 732600, and 625975. Copies of this information may be obtained free of charge from the Director, CCDC, 12 Union Road, Cambridge CB2, IEZ, UK (Fax: +44-1223-336-033; E-Mail: deposit@ccdc.cam.ac.uk or http://ccdc.cam.ac.uk).
